# Comprehensive analysis of the association between tumor glycolysis and immune/inflammation function in breast cancer

**DOI:** 10.1186/s12967-020-02267-2

**Published:** 2020-02-18

**Authors:** Wenhui Li, Ming Xu, Yu Li, Ziwei Huang, Jun Zhou, Qiuyang Zhao, Kehao Le, Fang Dong, Cheng Wan, Pengfei Yi

**Affiliations:** 1 Department of Breast and Thyroid Surgery, Union Hospital, Tongji Medical College, Huazhong University of Science and Technology, Wuhan, 430022 China; 2 Department of Nephrology, Union Hospital, Tongji Medical College, Huazhong University of Science and Technology, Wuhan, 430022 China

**Keywords:** Breast cancer, Glycolysis, Immune/inflammation function, PGK1, IL-17 signaling pathway, TCGA, Prognosis

## Abstract

**Background:**

Metabolic reprogramming, immune evasion and tumor-promoting inflammation are three hallmarks of cancer that provide new perspectives for understanding the biology of cancer. We aimed to figure out the relationship of tumor glycolysis and immune/inflammation function in the context of breast cancer, which is significant for deeper understanding of the biology, treatment and prognosis of breast cancer.

**Methods:**

Using mRNA transcriptome data, tumor-infiltrating lymphocytes (TILs) maps based on digitized H&E-stained images and clinical information of breast cancer from The Cancer Genome Atlas projects (TCGA), we explored the expression and prognostic implications of glycolysis-related genes, as well as the enrichment scores and dual role of different immune/inflammation cells in the tumor microenvironment. The relationship between glycolysis activity and immune/inflammation function was studied by using the differential genes expression analysis, gene ontology (GO) analysis, Kyoto Encyclopedia of Genes and Genomes (KEGG) analysis, gene set enrichment analyses (GSEA) and correlation analysis.

**Results:**

Most glycolysis-related genes had higher expression in breast cancer compared to normal tissue. Higher phosphoglycerate kinase 1 (PGK1) expression was associated with poor prognosis. High glycolysis group had upregulated immune/inflammation-related genes expression, upregulated immune/inflammation pathways especially IL-17 signaling pathway, higher enrichment of multiple immune/inflammation cells such as Th2 cells and macrophages. However, high glycolysis group was associated with lower infiltration of tumor-killing immune cells such as NKT cells and higher immune checkpoints expression such as PD-L1, CTLA4, FOXP3 and IDO1.

**Conclusions:**

In conclusion, the enhanced glycolysis activity of breast cancer was associated with pro-tumor immunity. The interaction between tumor glycolysis and immune/inflammation function may be mediated through IL-17 signaling pathway.

## Background

Breast cancer is the leading cause of cancer death among women [1]. Breast cancer can be classified as Normal-like, Luminal A, Luminal B, HER2-enriched and Basal-like subtypes. In addition to surgery, systemic therapies including chemotherapy, hormonal therapy, and molecular targeted therapy can be chosen based on the molecular characteristics to combat cancer [2]. Although these systemic therapies have improved patients’ outcomes, many patients do not respond to these existing treatments, which leads to poor prognosis [3, 4]. On this account, some researches have been carried out to explore new effective therapies in breast cancer such as Immunotherapy and metabolic therapy [5, 6]. However, only a minority of patients benefit from these emerging therapies [7]. Exploring the interplay between tumor cells and tumor microenvironment could lead to deeper understanding of breast cancer initiation, progression, and therapeutic resistance, possibly provide prospective strategies for cancer prevention and treatment [8, 9].

Metabolic reprogramming is a key hallmark of cancer [10]. The most frequently mentioned way of metabolic reprogramming is aerobic glycolysis. Aerobic glycolysis, also known as the “Warburg effect”, is a general way of glucose metabolism in cancer cells. In this way, glucose is mainly processed into lactate even when oxidative capacity is intact. This will lead to a highly acidic microenvironment. According to current research, tumor aerobic glycolysis can contribute to malignant transformation and tumor progression [11]. Therefore, tumor aerobic glycolysis has possible implications for prognosis judgment and cancer treatment [12]. Exploiting tumor glycolysis for clinical application requires figuring out how intrinsic and extrinsic factors to be integrated to modify the metabolic phenotype [13].

Tumor microenvironment (TME), cancer cells’ supporting hotbed, has complicated and changeable composition including tumor infiltrating lymphocytes (TILs), other immune and inflammatory cells, fibroblasts, the blood and lymphatic vascular networks, the extracellular matrix (ECM) and so on [14]. Numerous evidences suggest that the immune cells infiltration in the TME could interact with tumor cells, which may affect tumor progression and the efficacy of existing anticancer therapies [15, 16]. The immune cells recruited to the tumor site have dual characters, some can restrain carcinogenesis and tumor progression while others may play a tumor-promoting role [17]. Thus, it is important to figure out the cellular heterogeneity composition of the immune cells infiltration and the cause behind it. The results may be significant for optimizing existing treatments and identifying novel therapeutic targets [18].

Several studies have investigated the relationship between tumor glycolysis and immune/inflammation function [19–21]. A highly acidic microenvironment due to tumor glycolysis may differentially influence immune cells infiltration, ultimately leading to immune escape and cancer progression [22]. Exploration of the associations is providing a deeper look into cancer biological processes and can lead to more effective therapy selection. So far, however, there has been little comprehensive analysis focusing on the relationships between the tumor glycolysis, immune/inflammation function and the clinical features based on clinical data in the field of breast cancer. Given this, we implemented studies with transcriptome and clinical data of breast cancer from The Cancer Genome Atlas (TCGA) projects to explore the landscape of tumor glycolysis and immunity in breast cancer, to identify the relationship between the tumor glycolysis and immune and inflammatory cells infiltration, and to figure out the impact of the two on breast cancer prognosis.

## Methods

### The preprocessing step for transcriptome and clinical data

We obtained mRNA transcriptome data of breast cancer from TCGA. The RNA sequence data of breast cancer was downloaded directly from TCGA data portal (https://portal.gdc.cancer.gov/). The obtained expression data of 19657 genes from 1208 samples including 112 normal tissues and 1096 tumor tissues were used for follow-up research. The corresponding clinicopathological features including ER, PR, Her2 status, tumor stage, survival time and survival status were downloaded from TCGA data portal.

### Identification of differentially expressed genes and functional enrichment analysis

The tumor samples were divided into low glycolysis group and high glycolysis group based on the median expression of PGK1. Gene expression profiles were derived based on differential genes expression analysis using limma R package. Differentially expressed genes (DEGs) were identified through volcano plot filtering. The thresholds for DEGs were |log2FC| ≥ 1 and adjusted P value < 0.05. Gene ontology (GO) analysis and Kyoto Encyclopedia of Genes and Genomes (KEGG) analysis were performed using DAVID (https://david.ncifcrf.gov/) and WebGestalt (http://www.webgestalt.org/) [23, 24], using P < 0.05 as the cut-off criterion. Gene set enrichment analysis (GSEA) was conducted to find out the significantly upregulated and downregulated pathways between high and low glycolysis group using WebGestalt, using FDR < 0.05 as the cut-off criterion.

### Cell type enrichment analysis

To evaluate the cellular heterogeneity of the tumor microenvironment, the xCell tool, a gene signature-based method, was used to figure out the abundance of cell types in tumor microenvironment, based on the tissue transcriptome profiles [25]. Total 64 cell types including adaptive and innate immune cells, stem cells, epithelial cells and extracellular matrix cells and their enrichment scores were obtained. The present study focused on immune landscape of breast cancer. Therefore, 34 adaptive and innate immune cell types were retained for the further analysis.

### TIL density analysis

Mappings of tumor-infiltrating lymphocytes (TILs), based on digital scanned H&E images from TCGA breast cancer samples were downloaded from The Cancer Imaging Archive (TCIA) [26]. TIL maps were identified by a convolutional neural network trained computational stain developed by Saltz et al. [27]. Spearman correlation coefficient of glycolysis and TIL percentage from spatial estimates of TIL maps was calculated.

### Statistical analysis

SPSS 23.0 and R 3.4.4 were used for statistical analysis. Differences of continuous data between groups were analyzed by the ANOVA test. Categorical data were compared using the χ^2^ or Fisher exact test. Spearman correlation analysis was used to assess the potential relevance. Overall survival rate of each group was estimated using the Kaplan–Meier method and differences between groups were assessed by using the log-rank test. Cox proportional hazard regression model was used to analyze the prognostic significance of glycolysis-related genes. P value < 0.05 was considered statistically significant.

## Results

### Expression of glycolysis-related genes and the prognostic significance

**Fig. 1:**
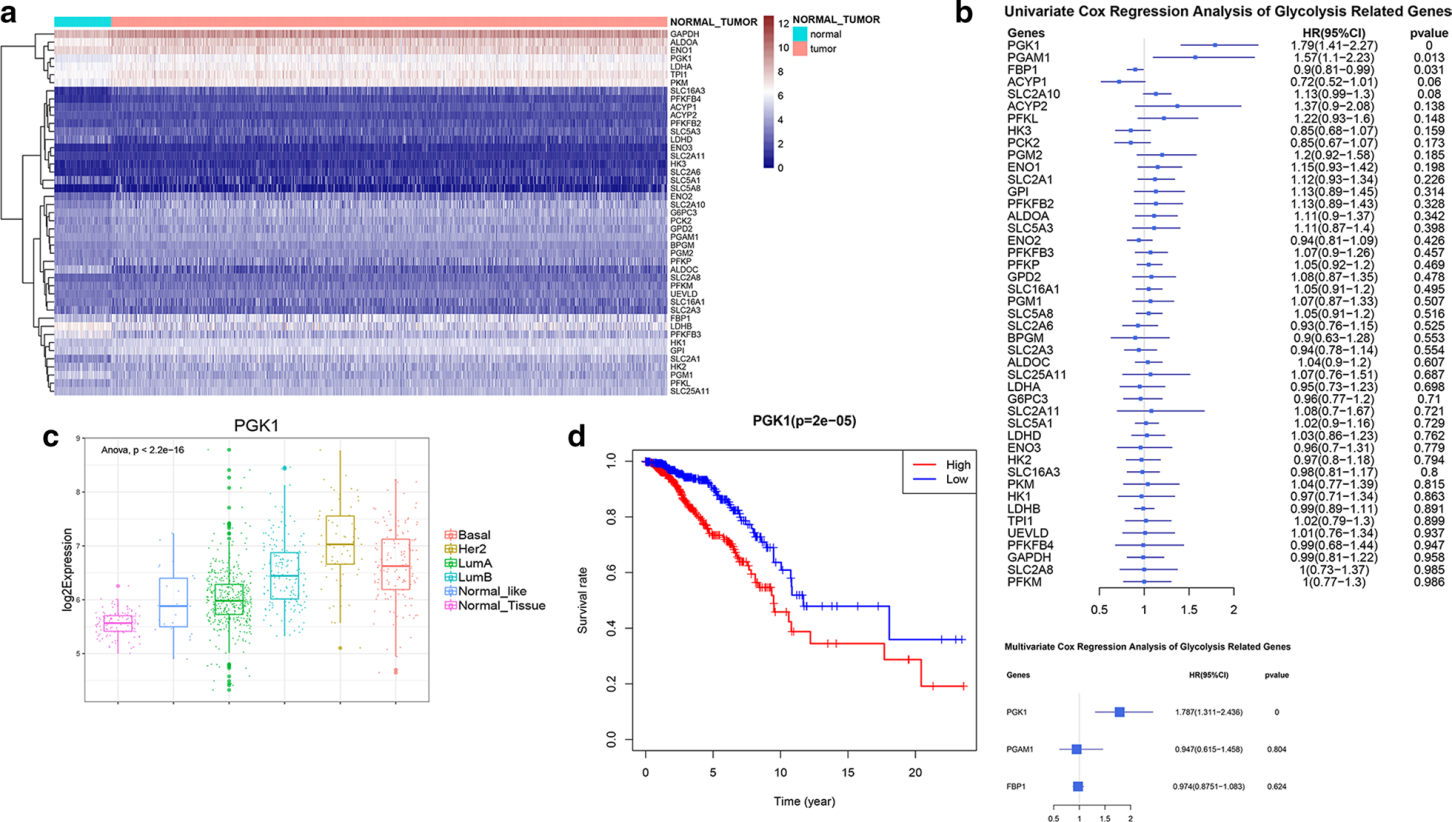
Expression of glycolysis-related genes and its prognostic significance. **a** Expression of glycolysis and gluconeogenesis related genes in normal and tumor tissues. **b** Univariate Cox regression and multivariate Cox regression were applied to calculate hazard ratios (HRs) of glycolysis-related genes. **c** The boxplot showed expression of PGK1 in normal tissues and different subtypes of breast cancer. **d** Kaplan–Meier survival analysis of low and high PGK1 expression was shown

The catabolic glycolysis and anabolic gluconeogenesis are two reciprocal pathways of glucose metabolism, which are catalyzed by a series of enzymes. Referring to published literature, 45 glycolysis and gluconeogenesis related genes, which can encode enzymes and metabolite transporters in the glucose metabolism pathways, were selected [28]. We compared the expression profile of these glycolysis and gluconeogenesis related genes between normal mammary tissues and breast cancer tissues. A heatmap was illustrated to display the results (Fig. 1a). The results revealed that the expression of 29 glycolysis and gluconeogenesis related genes including SLC2A1, HK2, GPI, PFKP, ALDOA, TPI1, GAPDH, PGK1, PGAM1, ENO1, PKM, LDHA, etc. were significantly higher in tumor tissues compared with normal mammary tissues. This result was consistent with the finding that tumors are characterized by enhanced glycolysis activity and glucose uptake for energy production and macromolecular biosynthesis [29].

Trying to screen out the candidate enzymes and metabolite transporters in glucose metabolism pathway that may play key roles in breast cancer progression, we explored the association between the expression of glycolysis and gluconeogenesis related genes and clinical manifestation of breast cancer based on TCGA database. Univariate and multivariate Cox proportional-hazards regression analysis were conducted to evaluate gene-associated hazard ratios (HRs) for overall survival. The results indicated that PGK1 overexpression was an independent risk factor for overall death in breast cancer (Fig. 1b).

The 1096 breast cancer patients derived from TCGA were then divided into two groups based on the median expression of PGK1. Higher PGK1 expression was positively related to larger tumor size, higher risk of lymphatic and distant metastasis, more advanced tumor stage and higher overall death. Moreover, the patients with higher PGK1 expression had a lower positive rate of ER and PR, but a higher positive rate of Her2 (Table 1). Compared with Luminal A and Luminal B subtypes, HER2-enriched and Basal-like subtypes had remarkable higher PGK1 expression (Fig. 1c). Survival analysis showed high PGK1 expression group was significantly associated with shorter overall survival, which confirmed the prognostic significance of PGK1 (Fig. 1d).

**Table 1:** Clinical characteristics of 1096 samples according to PGK1 low or high expression

Characteristics	PGK1		
	Low expression	High expression	P-value
Age	57.84 ± 12.81	58.77 ± 13.57	0.243
T stage			0.002
T1	156	124	
T2	304	331	
T3	77	62	
T4	10	29	
Tx	1	2	
N stage			0.047
N0	270	247	
N1	187	176	
N2	44	75	
N3	38	39	
Nx	9	11	
M stage			0.007
M0	453	457	
M1	4	17	
Mx	91	74	
Tumor stage (AJCC 8th version)			0.001
Stage I	110	72	
Stage II	309	315	
Stage III	117	131	
Stage IV	3	16	
Stage X	6	6	
NA	3	8	
ER status			< 0.001
Positive	461	345	
Negative	64	176	
NA	23	27	
PR status			< 0.001
Positive	410	288	
Negative	113	232	
NA	25	28	
Her2 status			< 0.001
Positive	50	111	
Negative	288	276	
NA	210	161	
Overall survival			< 0.001
Alive	495	453	
Dead	53	95	

### PGK1 expression can reflect the activity of glycolysis to some extent

**Fig. 2:**
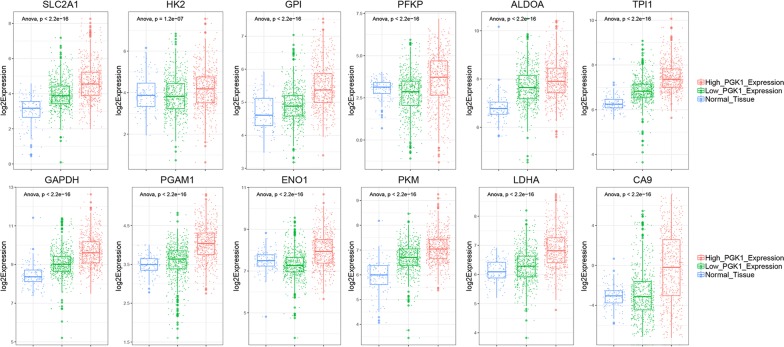
PGK1 expression can reflect the activity of glycolysis to some extent. The boxplots represented the expression of glycolytic pathway-specific genes SLC2A1, LDHA, ENO1, PFKP, PGAM1, PKM, HK2, GPI, TPI1, ALDOA, GAPDH and pH-regulated gene CA9 in normal tissue, low PGK1 expression and high PGK1 expression group

Phosphoglycerate kinase 1 (PGK1), the first ATP-generating enzyme in glycolytic pathway, catalyzes the conversion process from 1,3-diphosphoglycerate to 3-phosphoglycerate and from ADP to ATP. Given the important role of PGK1 in the glycolytic pathway and breast cancer progression, we selected PGK1 for further study. We then explored whether the expression of PGK1 could represent the activity of glycolysis. For this purpose, we compared the expression of other glycolysis related genes between normal tissues, low PGK1 expression group and high PGK1 expression group. The results showed that key genes in the glycolytic pathway including SLC2A1, HK2, GPI, PFKP, ALDOA, TPI1, GAPDH, PGAM1, ENO1, PKM, LDHA had remarkable higher expression in high PGK1 expression group (Fig. 2). What’s more, the expression level of carbonic anhydrase 9 (CA9) in high PGK1 expression group was seven times higher compared with low PGK1 expression group (Fig. 2). CA9 catalyzes the reversible hydration of carbon dioxide and participates in pH regulation. CA9 overexpression could prevent the accumulation of acidic metabolites in tumor cells at the expense of acidifying the extracellular tumor microenvironment. Thus, the overexpression of CA9 suggested higher glycolysis activity, lactic acid production and lactic acid output [30]. All the results above indicated that PGK1 expression can reflect the activity of glycolysis to some extent, therefore, low PGK1 expression group and high PGK1 expression group could be considered as low glycolysis group and high glycolysis group, respectively.

### Glycolysis activity was closely related with immune function

**Fig. 3:**
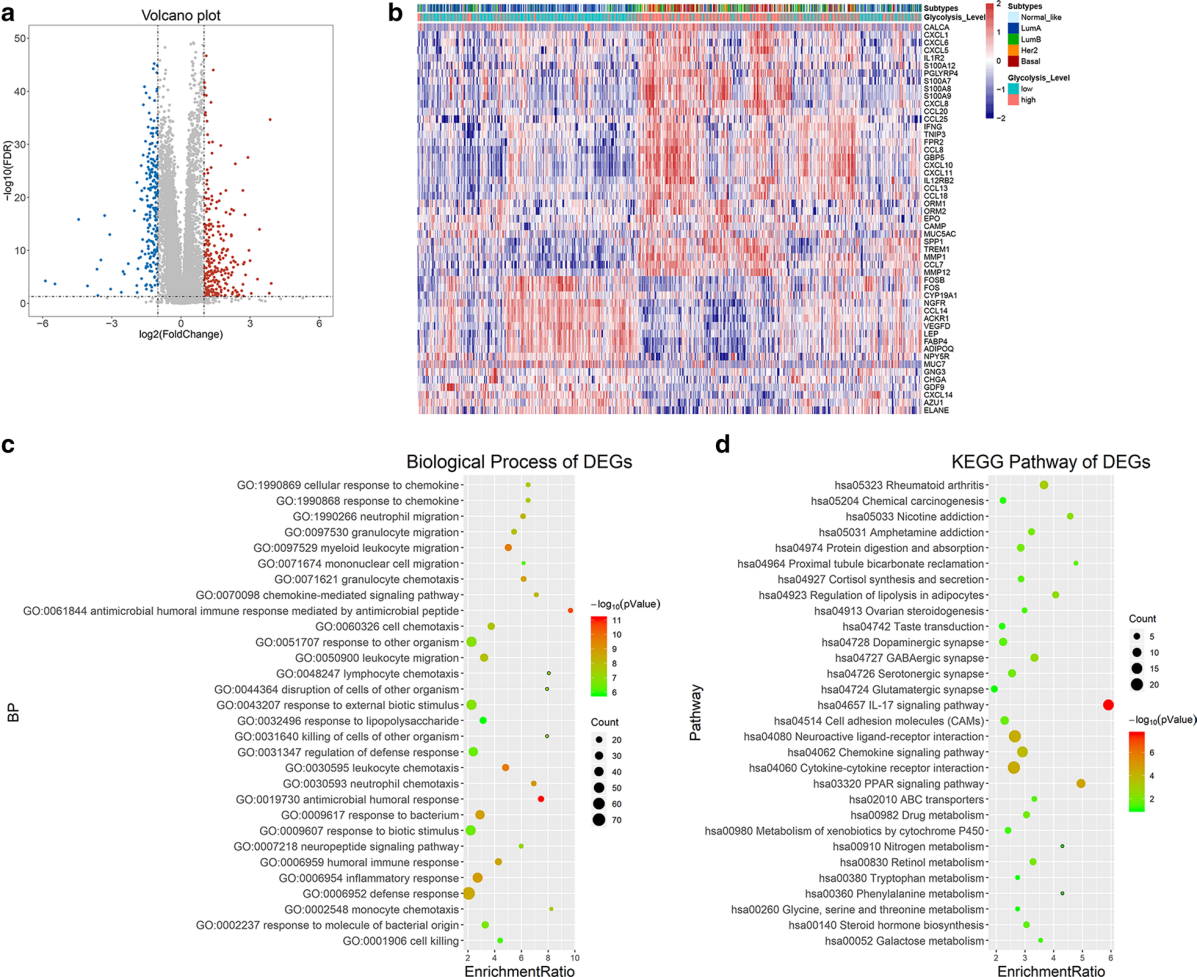
Glycolysis activity was closely related with immune function. **a** The volcano plot showed the DEGs between low glycolysis group and high glycolysis group. The blue color represented the downregulated genes, while the red showed the upregulated genes. **b** The heatmap displayed dysregulated inflammation-related genes, the glycolysis group and molecular subtypes group at the top of the heatmap. **c**, **d** GO and KEGG enrichment analysis of 470 DEGs. The top 30 most significant GO and KEGG terms were illustrated

To further investigate the biological difference between low glycolysis group and high glycolysis group, we analyzed the differential gene profiles between them 470 DEGs including 255 upregulated and 215 downregulated genes were identified to be significantly associated with the glycolysis activity (Fig. 3a). We found that numerous cytokines, chemokines, inflammatory mediators and related receptors were dysregulated in the high glycolysis group and involved in immune cell recruitment and function (Fig. 3b).

GO and KEGG enrichment analysis were conducted to uncover the gene function and biological pathways of DEGs. The results showed that immune-related functions were noticeable as shown in Fig. 3c, d. More specifically, the immune-related functions included GO:0061844 antimicrobial humoral immune response mediated by antimicrobial peptide, GO:0002548 monocyte chemotaxis, GO:0048247 lymphocyte chemotaxis, GO:0070098 chemokine-mediated signaling pathway, GO:0030593 neutrophil chemotaxis, GO:0071621 granulocyte chemotaxis, GO:0071674 mononuclear cell migration, GO:1990266 neutrophil migration, GO:0097530 granulocyte migration, GO:0097529 myeloid leukocyte migration, GO:0030595 leukocyte chemotaxis, GO:0050900 leukocyte migration, GO:0006954 inflammatory response, etc. (Fig. 3c). The enriched signaling pathways were hsa04657 IL-17 signaling pathway, hsa04060: Cytokine-cytokine receptor interaction, hsa04062: Chemokine signaling pathway and some metabolism-related signaling pathways (Fig. 3d).

### Immune-related pathways were upregulated in the high glycolysis group

**Fig. 4:**
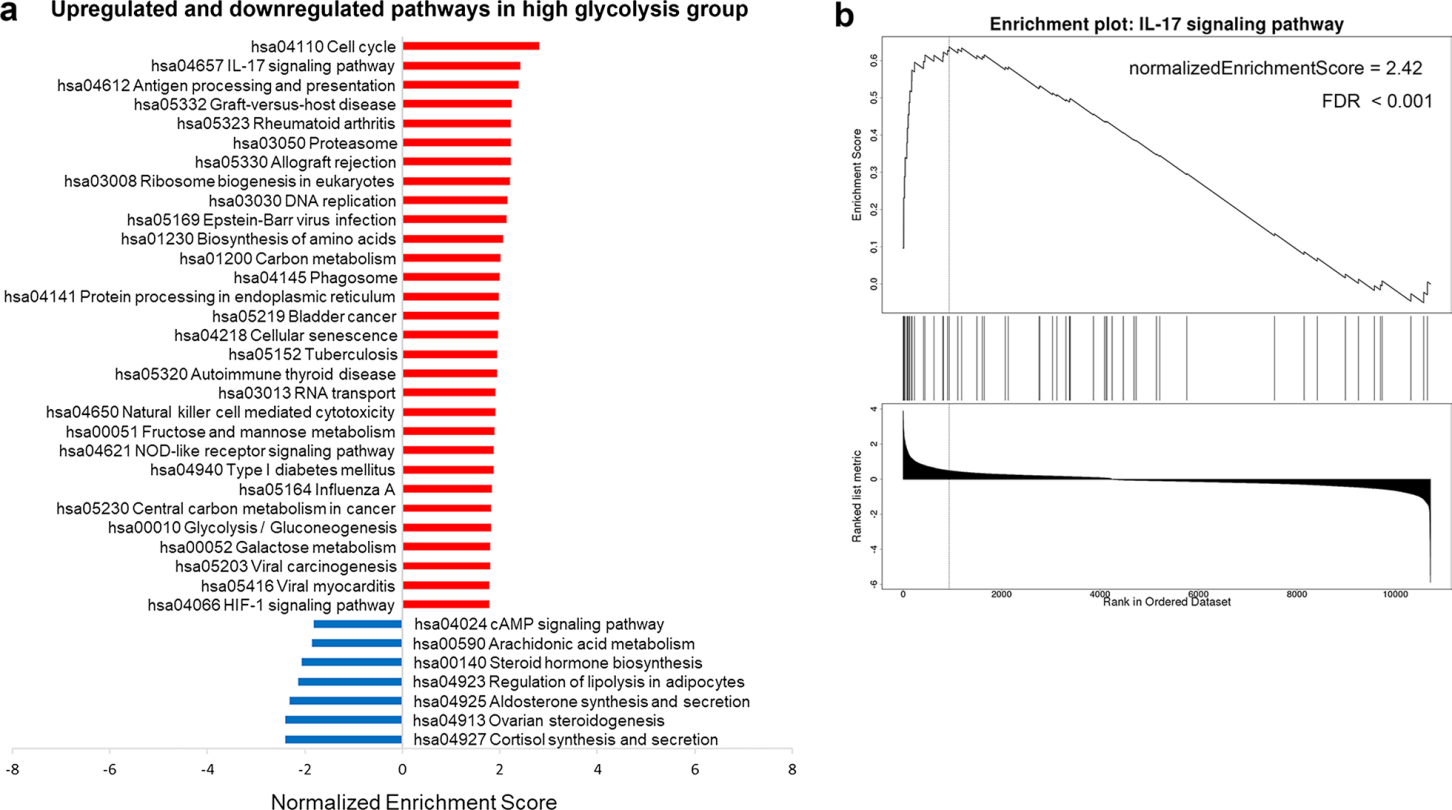
Immune-related pathways were upregulated in the high glycolysis group. **a** Top 30 upregulated pathways and seven downregulated pathways in high glycolysis group were obtained by GSEA. The red bars represented the upregulated pathways, while the blue bars showed the downregulated pathways. **b** GSEA showed that IL-17 signaling pathway was significantly upregulated in high glycolysis group. The value of normalizedEnrichmentScore and FDR were displayed in the figure

Given the finding above, we then conducted GSEA to explore whether immune-related pathways were upregulated or downregulated in high glycolysis group. FDR < 0.05 was considered statistically significant. Top 30 upregulated pathways and seven downregulated pathways were shown in Fig. 4a. The results revealed that numerous immune-related KEGG pathways were significantly upregulated, which included hsa04657 IL-17 signaling pathway (Fig. 4b), hsa04612 Antigen processing and presentation, hsa05332 Graft-versus-host disease, hsa05323 Rheumatoid arthritis, hsa05330 Allograft rejection, hsa05169 Epstein-Barr virus infection, hsa04650 Natural killer cell mediated cytotoxicity, hsa04621 NOD-like receptor signaling pathway, etc. Glucose metabolism pathways were also upregulated as expected. Not only the immune-related pathways and glucose metabolism pathways but also some other pathways such as cell cycle, DNA replication and ovarian steroidogenesis were influenced by glycolysis activity, which were shown in Fig. 4a. Altogether, these findings indicated that high glycolysis activity was associated with enhanced immune/inflammation activity in breast cancer.

### Correlation between immune/inflammation cells infiltration and glycolysis activity

**Fig. 5:**
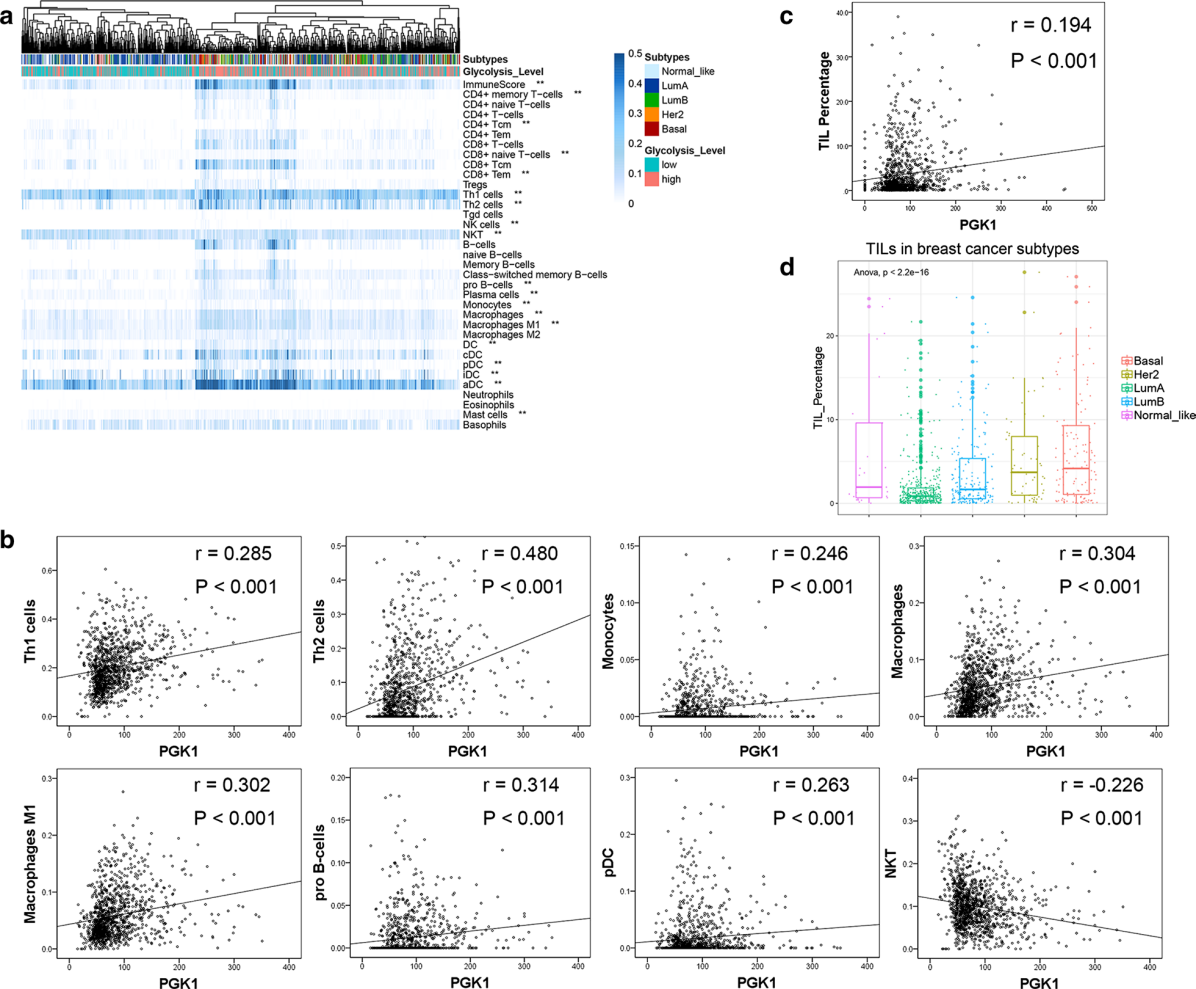
Correlation between immune/inflammation cells infiltration and glycolysis activity. **a** A heatmap showed the immune cell types enrichment scores of all samples. Groups based on glycolysis activity and molecular subtypes were shown for each sample (above the heatmap). Note that ** represented the cell types which showed significantly different enrichment between high and low glycolysis groups. **b** Correlation between PGK1 expression and individual immune cell enrichment scores were analyzed. The Spearman correlation coefficient between PGK1 expression and Th1 cells, Th2 cells, Monocytes, Macrophages, Macrophages M1, Pro B-cells, NKT cells were remarkable. **c** The scatter plot demonstrated that the Spearman correlation coefficient between PGK1 expression and TIL percentage. **d** TIL density in different subtypes of breast cancer was shown in the boxplot

In order to verify the association between immune/inflammation function and glycolysis activity, immune cell types enrichment scores were compared between low glycolysis group and high glycolysis group. High glycolysis group showed significantly higher immunescore. Most of the immune cell type enrichment scores were remarkable higher in high glycolysis group including CD4+ memory T-cells, CD8+ naive T-cells, CD8+ Tem, Th1 cells, Th2 cells, NK cells, pro B-cells, Plasma cells, Monocytes, Macrophages, Macrophages M1, DC, pDC, iDC and aDC. Instead, CD4+ Tcm, NKT cell and Mast cells enrichment scores were lower in high glycolysis group (Fig. 5a). What’s more, PGK1 expression was positively correlated with Th1 cells (*r* = 0.285, *P* < 0.001), Th2 cells (*r* = 0.480, *P* < 0.001), Monocytes (*r* = 0.246, *P* < 0.001), Macrophages (*r* = 0.304, *P* < 0.001), Macrophages M1 (*r* = 0.302, *P* < 0.001), Pro B-cells (*r* = 0.314, *P* < 0.001) and pDC (*r* = 0.263, *P* < 0.001), but negatively correlated with NKT cells (*r* = − 0.226, *P* < 0.001) (Fig. 5b). TILs density also showed positive correlation with tumor glycolysis (r = 0.194, P < 0.001) (Fig. 5c). HER2-enriched and Basal subtypes had the highest fraction of TILs, which was in line with PGK1 expression in breast cancer subtypes (Fig. 5d).

### High glycolysis may be associated with suppressed antitumor immunity

**Fig. 6:**
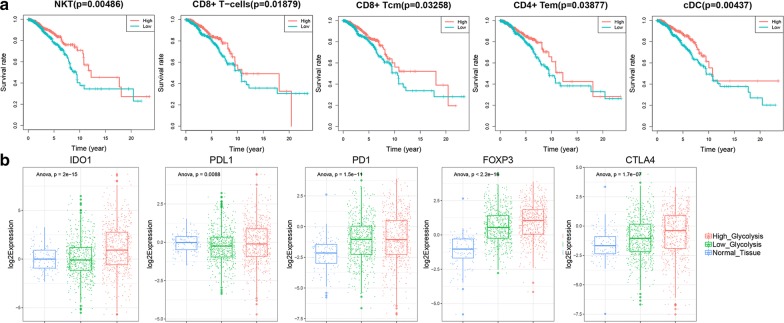
High glycolysis may be associated with suppressed antitumor immunity. **a** Survival analysis of each type of immune cell showed that groups with highly enriched cDC, NKT, CD8+ T-cells, CD8+ Tcm, CD4+ Tem cells had higher survival rates. **b** The expression of immunosuppressive factors including IDO1, PDL1, PD1, FOXP3 and CTLA4 in normal tissue, low glycolysis group and high glycolysis group

These immune and inflammatory cells may play a conflicting role in both tumor antagonism and tumor promotion. To identify the tumor-antagonizing and tumor-promoting immune and inflammatory cells, we conduct survival analysis to explore association between clinical outcome and enrichment scores of immune cell types in the tumor microenvironment. The results revealed that highly enriched cDC, NKT, CD8+ T-cells, CD8+ Tcm, CD4+ Tem cells led to better overall survival in breast cancer, while the immunescore and other immune cell types enrichment scores didn’t show prognosis significance (Fig. 6a). However, these immune cells which may play an antitumor role weren’t enriched in high glycolysis group. Instead, NKT cell enrichment scores were significantly lower in high glycolysis group.

Among the 255 upregulated genes, we found that the expression of Indoleamine 2,3-dioxygenase 1 (IDO1) in high glycolysis group is three times higher than that in low glycolysis group (Fig. 6b). IDO1 encodes an enzyme that catalyzes the rate-limiting step of tryptophan catabolism to the kynurenine and acts as a suppressor of anti-tumor immunity. Tryptophan shortage and kynurenine accumulation caused by IDO1 overexpression inhibit T lymphocytes proliferation and induce apoptosis of cytotoxic T lymphocytes (CTLs) [31, 32]. Based on this finding, we further compared the expression of other well-known immunosuppressive factors including PD1, PDL1, CTLA4 and FOXP3 [33]. Similar with IDO1, we found that PDL1, CTLA4 and FOXP3 also had higher expression in high glycolysis group (Fig. 6b). Overexpression of immunosuppressive factors and lack of NKT cells in high glycolysis group both indicated that high glycolysis may be associated with suppressed antitumor immunity.

## Discussion

Sufficient energy and metabolic intermediates for biosynthesis are the foundation of tumor cells initiation, proliferation and metastasis. Thus, many types of cancer are characterized by enhanced level of glycolysis and suppressed mitochondrial metabolism. In this way, tumor cells gain an advantage over stromal and immune cells in the competition to share limited nutrients and acquire ATP and intermediate metabolites faster [11]. The present study demonstrated that the key enzymes in glycolytic pathways had higher expression in breast cancer tissue, which indicated increased glycolysis activity in tumors. Among these glycolysis related genes, PGK1 has attracted our attention for its significant correlation with advanced tumor stage, and poor prognosis. By regulating ATP and 3-phosphoglycerate levels, PGK1 plays a critical role in coordinating energy production with biosynthesis and redox balance [34]. A recent comprehensive analysis of pan-cancer metabolic transformation also indicated that PGK1 was the most frequently upregulated gene in glycolytic pathway and had 100% frequency of upregulation in different types of cancer [28]. It has been demonstrated that high expression and activity of PGK1 was associated with poor prognosis in several types of cancer [35, 36]. Metabolism remodeling is an exquisite process regulated by oncogenic signaling pathways and transcription factors [11]. PGK1 functions as downstream targets of multiple oncogenic signal pathways and directly regulated by MYC,HIF-1α and other major regulator in metabolic reprogramming [37]. What’s more, PGK1 can translocate to Mitochondria and function as a protein kinase to inhibit pyruvate dehydrogenase complex, and promote tumor progress by increasing glycolysis and suppressing mitochondrial tricarboxylic acid cycle [38]. All of the above evidence indicated that PGK1 played a key role in metabolism reprogramming. Our study confirmed this hypothesis as glycolytic pathway-specific genes and pH-regulating genes had remarkable higher expression and glucose metabolism pathways and cell proliferation-related pathways such as cell cycle and DNA replication significantly upregulated in high PGK1 expression group. Therefore, PGK1 expression was regarded to represent the activity of glycolysis in the present study.

Much work so far has focused on the tumor glycolysis. However, the biological events affected by the tumor glycolysis, especially its immune/inflammation regulatory role, haven’t been well clarified. we compared gene expression profile, immune cell type enrichment scores and TIL percentage between low and high glycolysis groups and conducted functional enrichment analysis. The results suggested that numerous cytokines, chemokines, related receptors and immune/inflammation-related pathways were upregulated in the high glycolysis group. Consistent with this, high glycolysis group had a higher immunescore and higher TIL percentage. However, NKT, CD8+ T-cells, CD8+ Tcm, CD4+ Tem, cDC cells which may have an anti-tumor effect in breast cancer weren’t enriched in high glycolysis group. What’s more, immunosuppressive factors including IDO1, PD1, PDL1, CTLA4 and FOXP3 showed remarkable higher expression in high glycolysis group.

The upregulated immune factors and increased infiltration of immune cells did not bring a good outcome to the patients in the high glycolysis group. It seemed to be an inflammatory condition. Inflammation is another key hallmark for cancer initiation and progression, which refers to the local immune response involving cytokines, chemokines, small inflammatory protein mediators, and infiltrating immune cells in the TME [15]. The presence of tumor-antagonizing CTLs and NK cells is not surprising, however the list of tumor-promoting inflammatory cells now includes monocytes, macrophages, DCs, mast cells and neutrophils, as well as T and B lymphocytes [39]. Our study also indicated that high glycolysis contributed to an immunosuppressive tumor microenvironment. The phenomenon that tumor glycolysis and immune/inflammation function influence each other was also observed in other studies. One study found that IL-6 secreted by macrophages can phosphorylate PGK1 to promote glycolysis and tumorigenesis [40]. Several excellent studies have revealed that the metabolic microenvironment of tumor cells including glucose deprivation, lactic acid accumulation and acidification of tumor microenvironment may selectively disable tumor-killing T and NK cell activation and promote immune evasion, while attracting tumor-promoting inflammatory cells [19, 22, 41–45]. Lactic acid accumulation was demonstrated to induce macrophages toward an inflammatory pro-tumor phenotype [46]. In addition to the one-way effect, some studies have shown that cytokines from inflammatory cells and lactic acid from tumor cells can form a positive feedback loop to promote tumor progression [20, 47].

In our study, the most enriched immune/inflammation-related pathway in high glycolysis group was IL-17 signaling pathway. Emerging evidence indicated the tumor-promoting role of IL-17 in colorectal cancer, pancreatic cancer and lung cancer [48–50]. The upregulation of IL-17 signaling pathway recruited myeloid suppressive cells, which could induce angiogenesis and restrain anti-tumor immunity [51]. Interestingly enough one study showed that increased glucose uptake and metabolism reprogramming mediated by IL-17 signaling pathway was necessary for the activation of lymph node stromal cells [52]. Our study revealed that IL-17 mediated metabolism reprogramming existed not only in lymph node stromal cells but also in breast cancer tissues. In addition to macrophages which have been the research focus of pro-tumor inflammation, T helper cells especially Th2 cells showed remarkable correlation with tumor glycolysis in our study (*r* = 0.480, *P* < 0.001). Previous studies indicated that tumor-associated antigen and HLA-G expressed in tumors can promote immune evasion by strongly favoring Th2 development [53, 54]. However, the relationships between Th2 cells and tumor glycolysis have not been elucidated. Since our study was entirely based on public database and bioinformatic methods, the results should be interpreted with caution. Further in vitro and in vivo studies are needed to confirm our point.

Taken together, these evidences indicated that tumor glycolysis and pro-tumor immunity interdependently promoted each other. Thus, targeting glycolysis may refresh anti-tumor immunity and improve responsiveness to existing treatment such as chemotherapy and Her2-targeted therapy [55]. Since high glycolysis group had higher expression of immune checkpoint and higher TILs density which were both promising predictor for immunotherapy [56], they may exhibit a better immunotherapy response. In line with our research, tumor metabolic parameters on fluorodeoxyglucose positron emission tomography (FDG-PET) showed positive correlation with PD1/PDL1 expression and presence of TILs in non-small lung cancer [57, 58]. Some literature have also suggested the value of FDG-PET in predicting response to immune checkpoint inhibitors in non-small cell lung cancer [59], which partially supported our hypotheses. In our study, Her2-enriched and basal subtypes had higher glycolysis activity and higher immune inflammation cells infiltration in our study, which was consistent with its higher proliferative ability and higher malignant potential [2]. Thus, FDG-PET as the noninvasive detection method of tumor glycolysis may have the potential value to identify molecular subtypes of breast cancer and predict response rate to neoadjuvant chemotherapy since TIL is already known as a good predictor of response to neoadjuvant chemotherapy [60–62].

Given the critical role of PGK1 in metabolism remodeling and tumorigenesis, it may be a potential therapeutic target. However, targeting key metabolic enzyme can lead to severe systemic toxicity, because they are also essential for sustaining the normal cell function [6]. Attenuating the enzymatic activity of PGK1 by targeting its post-translational modifications (including phosphorylation, acetylation, ubiquitination, etc.) which have been shown significantly altered in cancer might reduce systemic toxicity and is an alternative approach [35, 40, 63, 64]. Despite all this, there is an urgent requirement for clinical application to establish a selective targeted delivery system to inhibit PGK1 activity in cancer cells precisely [6].

Numerous studies have revealed that the initiation and progression of cancer are dependent on a complex interplay between tumor cells and tumor microenvironment. Our study demonstrated that tumor glycolysis and immune inflammatory response may be the entry points for exploring the communication between tumor cells and tumor microenvironment. Deeper insight of the correlation between tumor glycolysis and immune inflammatory infiltration may lead to a revolution in cancer prevention and treatment.

## Conclusion

In conclusion, the enhanced glycolysis activity of breast cancer was associated with enriched inflammatory cell infiltration and enhanced inflammation response but higher expression of immunosuppressive factors and inhibition of antitumor immunity. The interaction between tumor glycolysis and immune/inflammation function may be mediated through IL-17 signaling pathway. Thus, drugs targeting metabolism may have an anti-inflammatory effect and vice versa. The level of glycolysis activity may guide the selection of checkpoint inhibitor immunotherapy. PGK1, as an important enzyme in glycolytic pathways, had obvious influence on breast cancer patients’ clinical outcome, which revealed that PGK1 played a critical role in the progression of breast cancer and may be a potential therapeutic target.

## Data Availability

All data was obtained from The Cancer Genome Atlas (TCGA, https://tcgadata.nci.nih.gov/tcga/). Other data generated or analyzed during this study were included in this published article and its additional files.

## Abbreviations

TME: Tumor microenvironment
TILs: Tumor infiltrating lymphocytes
ECM: Extracellular matrix
TCGA: The Cancer Genome Atlas
DEGs: Differentially expressed genes
GSEA: Gene set enrichment analysis
TCIA: The Cancer Imaging Archive
HRs: Hazard ratios
PGK1: Phosphoglycerate kinase 1
CA9: Carbonic anhydrase 9
IDO1: Indoleamine 2,3-dioxygenase 1
CTLs: Cytotoxic T lymphocytes
FDG-PET: Fluorodeoxyglucose positron emission tomography
